# FROM THESE ROOTS: THE ROLE OF HBCUS IN PREPARING THE NATION’S GERONTOLOGISTS FOR AN AGING SOCIETY

**DOI:** 10.1093/geroni/igad104.0510

**Published:** 2023-12-21

**Authors:** Terrell Brown

**Affiliations:** Jackson State University, Jackson, Mississippi, United States

## Abstract

The aging of the U.S. population is creating an increased need for social workers and other helping professionals with training in gerontology. Estimates indicate that less than 3% of MSW students are enrolled in an aging concentration, as compared to 19% enrolled in children/youth concentrations. The phenomenon of a diverse baby boomer generation joining the ranks of persons age 65 and older has created a plethora of scholarship and curriculum development aimed at readying the aging network for the unprecedented growth of older persons. Social work can make unique contributions to the field of gerontology. This study asserts, however, that social work is not adequately prepared to practice in an increasingly diverse aging society. The social work profession has articulated commitments to acknowledging and affirming how diversity and culture shape the human experience and to developing social workers who can competently engage in research-informed practice and practice-informed research. However, there remains a need in social work education for more widespread use of culturally relevant pedagogies that can help achieve these goals. Informed by both the Afrocentric and Black perspectives, this study presents a content analysis of the curricula infused at two historically Black universities. The nature and extent of the contributions of historically Black universities to social work education is the focus of this article. The scope of this investigation also includes the identification of prominent Black social work educators and discusses the implications of these perspectives for more culturally informed gerontology curricula that promote culturally competent gerontological social workers

